# Supplementary material for: ProteinShader: illustrative rendering of macromolecules
Source: BMC Struct Biol. 2009 Mar 30;9:19. doi: 10.1186/1472-6807-9-19 (PMC2672931; doi:10.1186/1472-6807-9-19)
Supplement: Additional file 1 — ProteinShader program without source code. This compressed file contains the complete ProteinShader program including associated libraries, but no source code. A README.txt file gives an overview of the ProteinShader distribution, and the index.html file in the help subdirectory has directions on getting started with the program as well as a set of tutorials. [file 1472-6807-9-19-S1.zip › ProteinShader-beta-0_9_4-binary/help/api/org/proteinshader/structure/visitor/AminoAcidLabelVisitor.html]

AminoAcidLabelVisitor (ProteinShader API)


|  |  |  |
| --- | --- | --- |
| **Overview** \| **Package** \| **Class** \| **Use** \| **Tree** \| **Deprecated** \| **Index** \| **Help** | |  |
| PREV CLASS   **NEXT CLASS** | **FRAMES**    **NO FRAMES**     **All Classes** |  |
| SUMMARY: NESTED \| FIELD \| CONSTR \| METHOD | DETAIL: FIELD \| CONSTR \| METHOD |  |


---


## org.proteinshader.structure.visitor Class AminoAcidLabelVisitor

```
java.lang.Object
  org.proteinshader.structure.visitor.Visitor
      org.proteinshader.structure.visitor.AminoAcidLabelVisitor
```

---

``` public class AminoAcidLabelVisitor extends Visitor ```

Traverses the AminoAcids of a Structure and builds a non-redundant
list of residueIDs.

---

**Constructor Summary**

`AminoAcidLabelVisitor()`
  
          Constructs an AminoAcidLabelVisitor.


| **Method Summary** | |
| --- | --- |
| `void` | `clearList()`             Clears the list of labels. |
| `List<String>` | `getAminoAcidLabels()`             Returns the list of non-redundant labels (AminoAcid residueIDs) that |
| `void` | `visit(AminoAcid aminoAcid)`             Adds a residueID to the list of labels if it has not been seen before. |

**Methods inherited from class org.proteinshader.structure.visitor.Visitor**

`clear, includeAAHetAndWater, setDebug, setMode, setMode, setMode, setMode, setMode, setMode, setVisitSegments, visit, visit, visit, visit, visit, visit, visit, visit, visit, visit, visit`

**Methods inherited from class java.lang.Object**

`clone, equals, finalize, getClass, hashCode, notify, notifyAll, toString, wait, wait, wait`

**Constructor Detail**

### AminoAcidLabelVisitor

```
public AminoAcidLabelVisitor()
```

:   Constructs an AminoAcidLabelVisitor.


**Method Detail**

### clearList

```
public void clearList()
```

:   Clears the list of labels.

---


### visit

```
public void visit(AminoAcid aminoAcid)
           throws VisitorException
```

:   Adds a residueID to the list of labels if it has not been seen
    before.

    :   **Overrides:**: `visit` in class `Visitor`
    :   **Parameters:**: `aminoAcid` - the AminoAcid to get a label from. **Throws:**: `VisitorException` - if an error occurs during the traversal.

---


### getAminoAcidLabels

```
public List<String> getAminoAcidLabels()
```

:   Returns the list of non-redundant labels (AminoAcid residueIDs)
    that

    :   **Returns:**: The AminoAcid residueIDs as a list of Strings.


---


|  |  |  |
| --- | --- | --- |
| **Overview** \| **Package** \| **Class** \| **Use** \| **Tree** \| **Deprecated** \| **Index** \| **Help** | |  |
| PREV CLASS   **NEXT CLASS** | **FRAMES**    **NO FRAMES**     **All Classes** |  |
| SUMMARY: NESTED \| FIELD \| CONSTR \| METHOD | DETAIL: FIELD \| CONSTR \| METHOD |  |


---

# *Copyright © 2007-2008*
